# A Multiorgan Trafficking Circuit Provides Purifying Selection of Listeria monocytogenes Virulence Genes

**DOI:** 10.1128/mBio.02948-19

**Published:** 2019-12-17

**Authors:** Alexander Louie, Ting Zhang, Simone Becattini, Matthew K. Waldor, Daniel A. Portnoy

**Affiliations:** a Department of Molecular and Cell Biology, University of California, Berkeley, Berkeley, California, USA; b Division of Infectious Diseases, Brigham and Women’s Hospital, Harvard Medical School, Boston, Massachusetts, USA; c Department of Microbiology, Harvard Medical School, Boston, Massachusetts, USA; d Immunology Program, Sloan Kettering Institute, Memorial Sloan-Kettering Cancer Center, New York, New York, USA; e Howard Hughes Medical Institute, Boston, Massachusetts, USA; f Department of Plant and Microbial Biology, University of California, Berkeley, Berkeley, California, USA; University of Washington

**Keywords:** *Listeria monocytogenes*, STAMP, gastrointestinal infection, intracellular bacteria, pathogenesis

## Abstract

Listeria monocytogenes can cause a life-threatening illness when the foodborne pathogen spreads beyond the intestinal tract to distant organs. Many aspects of the intestinal phase of L. monocytogenes pathogenesis remain unknown. Here, we present a foodborne infection model using C57BL/6 mice that have been pretreated with streptomycin. In this model, as few as 100 L. monocytogenes CFU were required to cause self-limiting enterocolitis, and systemic dissemination followed previously reported routes. Using this model, we report that listeriolysin O (LLO) and actin assembly-inducing protein (ActA), two critical virulence determinants, were necessary for intestinal pathology and systemic spread but were dispensable for intestinal growth. Sequence tag-based analysis of microbial populations (STAMP) was used to investigate the within-host population dynamics of wild-type and LLO-deficient strains. The wild-type bacterial population experienced severe bottlenecks over the course of infection, and by 5 days, the intestinal population was highly enriched for bacteria originating from the gallbladder. In contrast, LLO-deficient strains did not efficiently disseminate and gain access to the gallbladder, and the intestinal population remained diverse. These findings suggest that systemic spread and establishment of a bacterial reservoir in the gallbladder imparts an intraspecies advantage in intestinal occupancy. Since intestinal L. monocytogenes is ultimately released into the environment, within-host population bottlenecks may provide purifying selection of virulence genes.

## Abstract (precis)

Listeria monocytogenes maintains capabilities for free-living growth in the environment and for intracellular replication in a wide range of hosts, including livestock and humans. Here, we characterized an enterocolitis model of foodborne L. monocytogenes infection. This work highlights a multiorgan trafficking circuit and reveals a fitness advantage for bacteria that successfully complete this cycle. Because virulence factors play critical roles in systemic dissemination and multiple bottlenecks occur as the bacterial population colonizes different tissue sites, this multiorgan trafficking circuit likely provides purifying selection of virulence genes. This study also serves as a foundation for future work using the L. monocytogenes-induced enterocolitis model to investigate the biology of L. monocytogenes in the intestinal environment.

## INTRODUCTION

The Gram-positive bacterium Listeria monocytogenes occupies a wide ecological niche that facilitates its entry into our food system. Upon ingestion, L. monocytogenes infects a broad range of hosts, including livestock and humans. In healthy adults, L. monocytogenes infection leads to self-limiting enterocolitis which usually does not require clinical attention, making accurate counts of L. monocytogenes infections difficult (1). In rare instances, L. monocytogenes leads to listeriosis, a systemic disease that often includes bacteremia and meningoencephalitis, and can lead to pregnancy loss (2). Risk factors for the development of listeriosis include compromised immunity and pregnancy. Although treatments are available, mortality rates remain near 25% (3).

The pathogenesis of L. monocytogenes depends on host cell entry, escape from the entry vacuole, intracellular replication, and spread to systemic sites. By secreting listeriolysin O (LLO), a pore-forming cytolysin encoded by the *hly* gene, L. monocytogenes disrupts the entry vacuole and enters the host cytosol where it replicates rapidly. Strains lacking LLO cannot access the host cytosol and thus fail to replicate intracellularly (4). Shortly after entering the host cytosol, actin assembly-inducing protein (ActA) decorates the bacterial surface and induces actin polymerization to propel the bacterium, first intracellularly and then to facilitate spread from one cell to the next. Strains lacking ActA replicate in the host cytosol but have a defect in spreading to adjacent cells. In mice, intravenous (i.v.) infection with strains lacking either LLO or ActA result in severely attenuated growth at sites of dissemination (4–8). Following initial infection of host cells in the intestinal epithelium, the pathogen eventually breaches the intestinal barrier and spreads to systemic sites in two waves. Within the first 24 h of infection, the first wave of bacteria arrives in the liver via the portal vein. The second wave spreads from mesenteric lymph nodes to the spleen. These two populations then intermix by exchange through the circulatory system (9). L. monocytogenes in the liver subsequently enters the gallbladder and replicates extracellularly to a high density (10). Upon bile excretion, L. monocytogenes reenters the intestinal tract and is shed in feces. Thus, during infection, the gallbladder can be transformed into a bacterial reservoir (11).

Both mice and humans are relatively resistant to orally acquired L. monocytogenes infections. Estimates suggest that the average person consumes L. monocytogenes-contaminated food 5 to 9 times a year, yet the reported incidence of listeriosis is orders of magnitude lower (12). Although the minimal infectious dose for L. monocytogenes in humans is unknown, a L. monocytogenes outbreak caused by contaminated chocolate milk suggested that a dose of approximately 3 × 10^11^ CFU led to the development of febrile gastroenteritis in healthy adults (13). Over the past few decades, the most widely studied animal model of L. monocytogenes pathogenesis has been the mouse i.v. infection model. The i.v. model approximates the more severe systemic form of the disease but completely bypasses the initial intestinal phase of the infection. Due in part to this limitation, L. monocytogenes biology within the intestinal tract remains incompletely defined. In C57BL/6 mice, administration of doses as high as 1 × 10^8^ CFU lead to L. monocytogenes recovered from feces and dissemination to systemic sites, but obvious signs of disease do not develop (14). These observations suggest that resistance mechanisms effectively control L. monocytogenes following ingestion. In other mouse models of disease caused by enteric pathogens, including Salmonella enterica serovar Typhimurium, Citrobacter rodentium, and Clostridium difficile, antibiotic treatment of mice prior to infection increases susceptibility to infection (15–17). Similarly, oral gavage of streptomycin 24 h prior to an oral gavage of L. monocytogenes dramatically enhances intestinal colonization (18).

Here, we describe a foodborne mouse model of L. monocytogenes-induced enterocolitis and find that the intracellular life cycle of L. monocytogenes is dispensable for growth in the intestinal tract but required for host pathology. Moreover, characterization of L. monocytogenes population dynamics in the intestinal tract revealed an intraspecies competition between intestine-resident and systemically derived bacteria. As the infection progressed, the majority of L. monocytogenes shed in feces originated from the gallbladder. Notably, this intraspecies competition did not arise in mice infected with an LLO-deficient strain, suggesting that this multiorgan trafficking circuit required LLO and that successful completion of the circuit imparts a fitness advantage. Collectively, our results demonstrate that the L. monocytogenes-induced enterocolitis model provides an exciting opportunity to study L. monocytogenes pathogenesis in the intestinal tract.

## RESULTS

### Streptomycin pretreatment increases susceptibility to foodborne Listeria monocytogenes infection.

**FIG 1:**
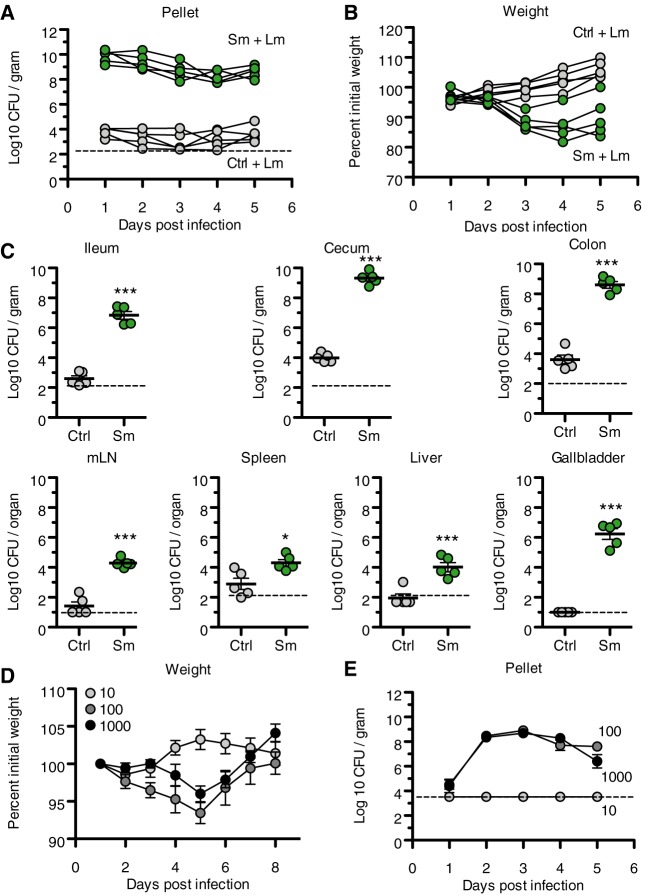
Streptomycin pretreatment enhances susceptibility of mice to foodborne L. monocytogenes (Lm) infection. (A) L. monocytogenes fecal shedding. C57BL/6 mice (*n* = 5) received either no treatment or streptomycin (Sm)-supplemented drinking water for 48 h prior to infection by voluntary consumption of bread containing 10^8^ L. monocytogenes CFU. Results are expressed as log-transformed CFU per gram of feces. Lines represent individual mice. Treatment: *P* < 0.001 (two-way analysis of variance [ANOVA]). (B) Body weight of mice in panel A. Results are expressed as a percentage of body weight prior to streptomycin treatment. Lines represent individual mice. Treatment: *P* < 0.001 (two-way ANOVA). (C) Bacterial burden 5 days postinfection of mice in panel A. Results are expressed as log-transformed means with standard errors. ***, *P* < 0.001 by unpaired two-sided *t* test. Dashed lines indicate limits of detection. (D) Body weights of mice receiving Sm and either 10, 100, or 1,000 L. monocytogenes CFU as described for panel A. Results are expressed as a percentage of body weight prior to streptomycin treatment, and means with standard errors are indicated. Two-way ANOVA, dose, *P* < 0.05; Bonferroni’s posttest, 10 versus 100, day 5: *P* < 0.001; 10 versus 1,000, day 5: *P* < 0.01). (E) L. monocytogenes fecal shedding in mice in panel D. Results are expressed as log-transformed means with standard errors. Two-way ANOVA, dose, *P* < 0.001; Bonferroni’s posttest, 10 versus 100, day 2: *P* < 0.001; 10 versus 1,000, day 2: *P* < 0.001). Dashed line indicates limit of detection. All results are representative of at least 2 independent experiments.

In our efforts to advance our understanding of L. monocytogenes pathogenesis, we modified a previously described foodborne model of L. monocytogenes infection where mice voluntarily consume a breadcrumb containing a defined number of bacteria (14). In contrast to oral gavage, this foodborne method of infection rules out possible injury during inoculation, which can lead to inadvertent systemic dissemination. One limitation of the foodborne method is the relatively high resistance of C57BL/6 mice to L. monocytogenes infection compared to that of other inbred mouse strains (14). Becattini et al. reported that antibiotic treatment of mice prior to infection dramatically increases susceptibility to orally acquired L. monocytogenes (18). We tested whether streptomycin (Sm) pretreatment affected foodborne L. monocytogenes infection in C57BL/6 mice. To noninvasively administer the antibiotic, we added 5 mg/ml streptomycin to the mouse drinking water 48 h prior to infection. Groups of Sm- and mock-treated female C57BL/6 mice were fasted overnight and individually fed a breadcrumb contaminated with 1 × 10^8^ CFU of the Sm-resistant L. monocytogenes strain 10403S. Immediately after the mice consumed the breadcrumb, they were returned to cages with *ad libitum* access to standard mouse chow and standard drinking water. To measure intestinal colonization, we enumerated L. monocytogenes CFU shed in the mouse feces over the course of 5 days (Fig. 1A). Relative to mock-treated mice, there was a million-fold increase in L. monocytogenes CFU from the Sm-pretreated mice (Sm mice). The Sm mice continued to shed upwards of 1 × 10^8^ CFU/gram during the 5-day observation period, whereas almost all of the mice in the mock-treated group remained just above the limit of detection. As a gross metric of disease severity, we monitored body weight over the course of infection (Fig. 1B). The weights of mice that received only Sm (see Fig. S1 in the supplemental material) and mice that were mock treated but infected with L. monocytogenes remained stable over the observation period. In contrast, Sm mice that received L. monocytogenes began to lose weight 3 days postinfection and lost ∼15% to 20% of their initial body weight by 4 days postinfection. At 5 days postinfection, Sm mice began to regain weight. In conjunction with weight loss, Sm mice also developed acute diarrhea reminiscent of the self-limiting gastroenteritis that L. monocytogenes causes in humans (see Fig. S2).

10.1128/mBio.02948-19.1 FIG S1 Body weights after streptomycin pretreatment. C57BL/6 mice (*n* = 5) received either no treatment (Ctrl) or streptomycin (Sm)-supplemented drinking water for 48 h. Download FIG S1, EPS file, 1.3 MB. Copyright © 2019 Louie et al. 2019 Louie et al. This content is distributed under the terms of the Creative Commons Attribution 4.0 International license.

10.1128/mBio.02948-19.2 FIG S2 Wet/dry ratio of fecal pellets. C57BL/6 mice (*n* = 5) received either no treatment (Ctrl) or streptomycin (Sm)-supplemented drinking water for 48 h prior to infection by voluntary consumption of bread containing 10^8^ L. monocytogenes CFU. Results are expressed as the log2-transformed ratio of wet and dry weights. *, *P* < 0.05; **, *P* < 0.01 by two-way ANOVA and Bonferroni’s posttest. Download FIG S2, EPS file, 1.3 MB. Copyright © 2019 Louie et al. 2019 Louie et al. This content is distributed under the terms of the Creative Commons Attribution 4.0 International license.

To examine the effect of streptomycin pretreatment on systemic dissemination after foodborne infection, mice were sacrificed 5 days postinfection and L. monocytogenes CFU were enumerated in the gastrointestinal tract, mesenteric lymph nodes, spleen, liver, and gallbladder (Fig. 1C). In all tissues examined, Sm mice contained significantly more CFU than mock-treated mice. Although previous studies of intestinal L. monocytogenes infections focused on the small intestine, the majority of recoverable L. monocytogenes CFU were found in the cecum and colon, where the pathogen burden exceeded that in control animals by ∼5 orders of magnitude. In stark contrast to the gallbladders from control mice, where L. monocytogenes was not detectable, all the gallbladders of Sm mice contained ∼10^6^ CFU (Fig. 1C).

To test if lower doses of L. monocytogenes also lead to disease, Sm mice were fed breadcrumbs containing 10, 100, or 1,000 CFU and monitored for changes in body weight and for shedding of L. monocytogenes in feces. The body weight of mice receiving 10 bacteria remained stable and L. monocytogenes was not detected in feces. However, inocula of only 100 bacteria led to weight loss during the course of infection (Fig. 1D). The amount of L. monocytogenes recovered from feces increased from ∼10^4^ CFU/g on day 1 to 1 × 10^9^ CFU/g by 3 days postinfection, indicating that L. monocytogenes robustly replicates within the streptomycin-pretreated intestines (Fig. 1E). Together, streptomycin pretreatment followed by foodborne infection with L. monocytogenes in C57BL/6 mice provide an excellent opportunity to examine the intestinal phase of L. monocytogenes pathogenesis in a genetically tractable host.

### Characterization of L. monocytogenes dissemination over the course of infection.

**FIG 2:**
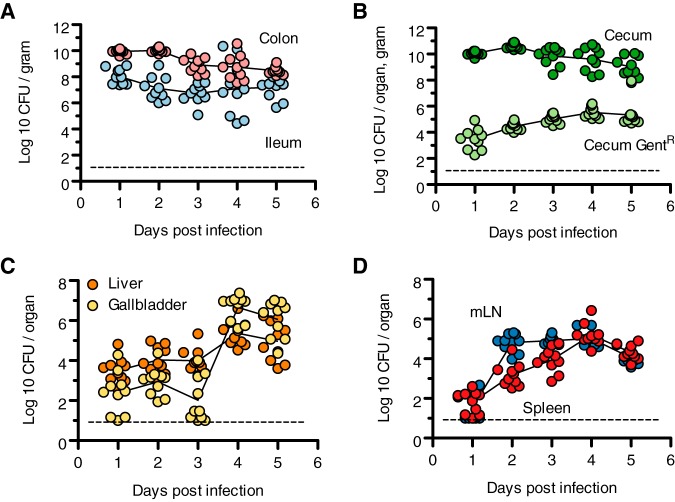
L. monocytogenes tissue colonization dynamics. C57BL/6 mice received Sm pretreatment and bread contaminated with 10^8^ L. monocytogenes CFU. Cohorts of mice (*n* = 10) were sacrificed at 24-h intervals, and bacterial burdens were determined. Results are expressed as log-transformed CFU per gram or organ as indicated, and data are combined from 2 independent experiments. (A) Colon and ileum CFU per gram of tissue. (B) Cecum CFU. Total L. monocytogenes CFU per gram and gentamicin-resistant L. monocytogenes CFU per organ are presented. (C) Liver and gallbladder CFU. (D) Mesenteric lymph node and spleen CFU. Dashed lines indicate limits of detection.

To evaluate the dynamics of L. monocytogenes systemic spread in this model, Sm mice were infected with 10^8^ CFU of foodborne L. monocytogenes, and cohorts of mice were sacrificed every 24 h to enumerate pathogen burden in the intestines, mesenteric lymph nodes, liver, gallbladder, and spleen. At 24 h postinfection, L. monocytogenes was recovered throughout the intestinal tract, with ∼10^8^ CFU/g in the ileum and ∼10^10^ CFU/g in the cecum and colon (Fig. 2A and B). The pathogen burden remained fairly stable over the course of 5 days. In intestinal tissue, the L. monocytogenes population was comprised of extracellular bacteria in the intestinal lumen and bacteria within host cells. To determine the fraction of intracellular bacteria, ceca were washed with phosphate-buffered saline (PBS) and treated with gentamicin prior to CFU enumeration, as gentamicin does not affect intracellular bacteria. We focused on the cecum due to the high abundance of bacteria. At 24 h postinfection, ∼10^4^ gentamicin-resistant CFU per organ were recovered from the cecum, indicating that intracellular bacteria represented a very minor fraction of the total pathogen burden in the intestine and suggesting that host cell entry occurred infrequently. Over 5 days, the intracellular population increased 10-fold (Fig. 2B).

L. monocytogenes were detectable at systemic sites as soon as 24 h postinfection. Among the systemic sites monitored, the liver had the highest bacterial burden at 24 h postinfection (∼10^4^ CFU per organ) (Fig. 2C and D), which is consistent with a model where the liver receives the first wave of bacteria via the portal vein (9). Because the gallbladder was previously reported to contain a reservoir of L. monocytogenes, we enumerated CFU in the gallbladder separately from the liver. During the first 3 days of infections, gallbladders contained roughly 10-fold fewer bacteria than the liver. However, by day 4, gallbladder CFU increased 10,000-fold, and bacterial burdens in the gallbladder surpassed those in the liver by ∼10-fold on days 4 and 5 postinfection.

The dynamics of L. monocytogenes dissemination to mesenteric lymph nodes (mLN) and spleen differed from those observed in the liver. One day postinfection, CFU in the mLN and spleen were 1,000-fold lower than in the liver (Fig. 2D). On day 2, CFU in the mLN increased 10,000-fold and plateaued over the course of the experiment. In contrast, we observed a steady ∼10-fold increase/day in the spleen over 4 days, but on day 5, a 10-fold decrease was observed. These observations are consistent with a second wave of L. monocytogenes dissemination from the mLN to the spleen as described in the guinea pig model (9). Taken together, the routes and dynamics of systemic spread in this model are consistent with previous reports (9, 11).

### LLO and ActA are required for intestinal pathology and systemic dissemination.

**FIG 3:**
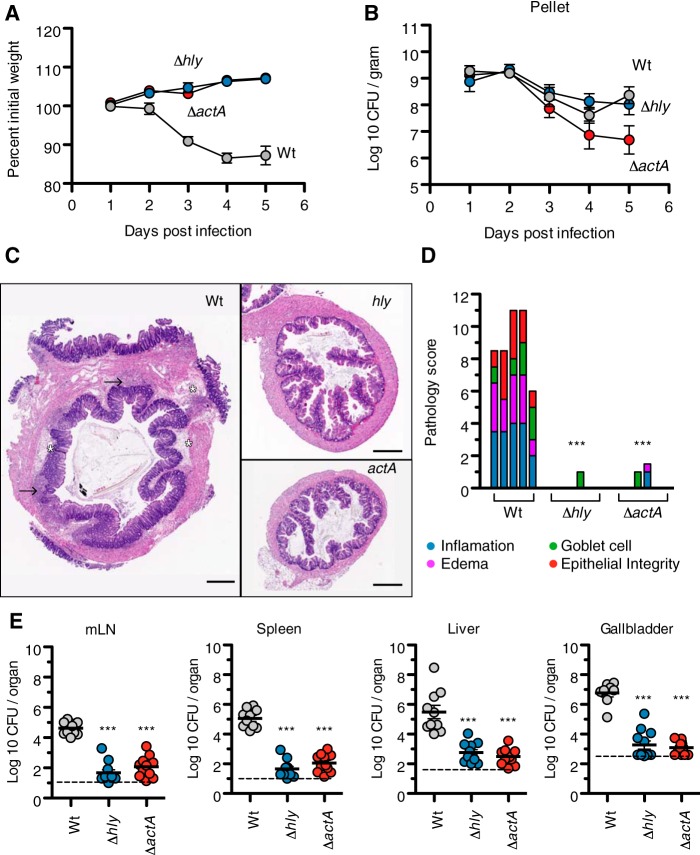
L. monocytogenes-induced intestinal pathology and systemic spread requires LLO and ActA. C57BL/6 mice received Sm pretreatment and bread contaminated with 10^8^ CFU of either wild-type, Δ*hly*, or Δ*actA* strains. (A) Body weights. Results are expressed as a percentage of body weight prior to streptomycin treatment. Two-way ANOVA and Bonferroni’s posttest, WT versus Δ*hly*, days 2 to 5: *P* < 0.05; WT versus Δ*actA*, days 2 to 5: *P* < 0.05 (B) Fecal shedding. Results are expressed as log-transformed means with standard errors. Two-way ANOVA and Bonferroni’s posttest, WT versus Δ*hly*, days 1 to 5: *P* > 0.05; WT versus Δ*actA*, day 5: *P* < 0.01. (C) Representative hematoxylin and eosin staining of colonic tissues. Scale bars, 500 μM. Sites of edema are indicated by asterisks, and sites of immune cell infiltrate are indicated by arrows. (D) Combined pathology scores. Bars represent individual animals with statistically significant differences compared to mice with the wild-type strain. ***, *P* < 0.001 by one-way ANOVA and Dunnett’s posttest. (E) Bacterial burdens. Results are expressed as log-transformed means with standard errors. ***, *P* < 0.001 versus mice infected with the wild type strain, one-way ANOVA and Dunnett’s posttest. Data are combined from two independent experiments.

We investigated the roles of two well-established and well-characterized virulence determinants, LLO and ActA, on the intestinal phase of L. monocytogenes pathogenesis. Groups of C57BL/6 mice were pretreated with streptomycin and infected with 10^8^ wild-type (WT), Δ*actA*, or Δ*hly* strains. In contrast to the WT strain, neither of the mutant strains led to weight loss in infected mice (Fig. 3A), even though the numbers of CFU of both mutant strains recovered in feces were similar to that for the WT for the first 3 days of infection (Fig. 3B). Compared to the WT strain, which caused goblet cell loss, submucosal edema, severe inflammation characterized by prominent multifocal submucosal cellular infiltrate, and loss of epithelial integrity marked by epithelial erosion and ulceration, the Δ*actA* or Δ*hly* strain caused little colonic pathology (Fig. 3C and D). Furthermore, the two mutants were markedly attenuated in their capacity to disseminate beyond the intestine (Fig. 3E). However, small numbers of bacteria were still recoverable from mesenteric lymph nodes, spleens, and livers of mice infected with either mutant strain, indicating that there are *hly*- and *actA*-independent mechanisms of spread. At least part of the reduced capacity of the Δ*hly* strain to spread and cause disease may be attributable to its diminished ability to enter or proliferate in intestinal cells; the quantity of intracellular bacteria (gentamicin-resistant CFU) in the ceca of animals infected with the Δ*hly* strain was 1,000-fold lower than observed with the WT strain (data not shown).

### Clonal enrichment of intestinal population depends upon systemic dissemination.

The observation that the *Δhly* strain did not have a detectable defect in fecal shedding led us to investigate how intracellular replication and systemic spread modifies the population dynamics of this enteric pathogen. As L. monocytogenes spreads systemically, a reservoir of extracellular bacteria develops in the gallbladder; these bacteria can then reenter the intestinal tract during bile excretion and be shed in the feces (10, 11). Given that the *Δhly* strain had a marked defect in accessing and/or proliferating in the gallbladder, we hypothesized that the *Δhly* strain would be unable to complete this within-host trafficking route, which could negatively impact fecal transmission in a natural setting. To explore this hypothesis, we generated a collection of 200 genetically tagged but otherwise isogenic strains of wild-type and Δ*hly* L. monocytogenes and tracked the presence and frequency of tags in the feces over the course of the infection and in the gallbladder at 5 days postinfection.

**FIG 4:**
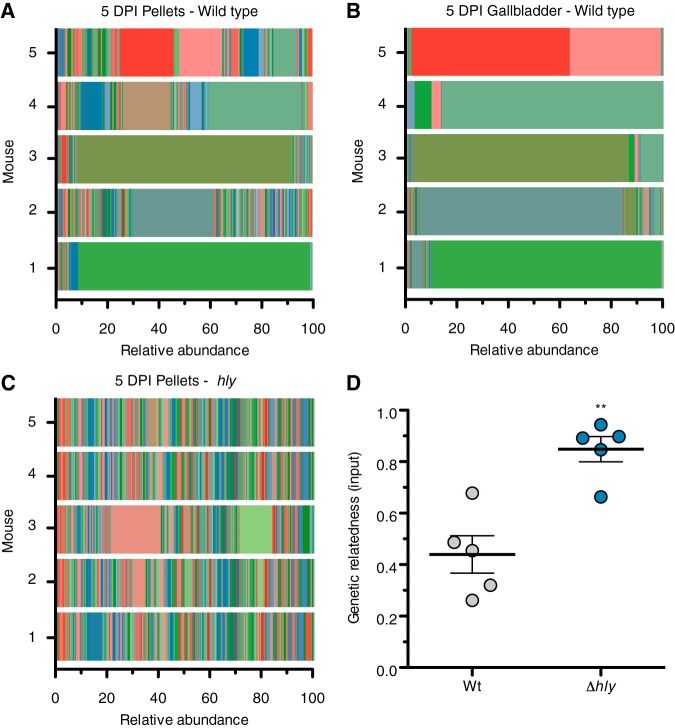
Within-host population dynamics of wild-type and Δ*hly* strains. C57BL/6 mice (*n* = 5) received Sm pretreatment and bread contaminated with 10^8^ CFU of a L. monocytogenes library containing 200 genetically tagged strains. Relative tag abundance from 5 days postinfection. Individual tags are indicated by different colors. (A) Fecal pellets recovered from mice infected with the wild-type strain. (B) Gallbladders. Same mice as in panel A. (C) Fecal pellets recovered from mice infected with a Δ*hly* strain. (D) Genetic relatedness of organisms recovered from fecal pellets 5 days postinfection relative to their respective inocula. A score of 1 indicates no divergence from the inocula. **, *P* < 0.01 by unpaired two-tailed *t* test.

In Sm mice infected with wild-type L. monocytogenes, the tag abundances recovered from fecal samples collected 1 and 2 days postinfection closely resembled the abundances found in the inoculum (see Fig. S3). By day 3, the fecal L. monocytogenes population structures began to deviate from the input population, which coincided with the onset of diarrhea and weight loss in the animal (Fig. 1B and S2). By day 5, we observed an enrichment of 1 or 2 tags in the fecal L. monocytogenes population (Fig. 4A and S3A). In two of five samples (mouse 1 and 3), ∼80% of the bacteria recovered from the feces shared the same genetic tag. In the remaining three samples, one or two tags accounted for approximately ∼40% of the bacterial population. Strikingly, the dominant tags found in the day-5 fecal populations matched the tags found in the gallbladder, which was predominantly populated with L. monocytogenes sharing 1 or 2 tags (Fig. 4A and B and S3A). The identity of fecal and gallbladder L. monocytogenes tags strongly suggested that by day 5 of infection, gallbladder-resident bacteria reentered and outcompeted the L. monocytogenes already present in the intestinal tract. Importantly, the identity of dominant tags recovered from feces and gallbladder differed between mice, ruling out the possibility that our tagged library contained a strain with improved host colonization. These observations are consistent with a previous study that used BALB/c mice and an InlA^m^ strain of L. monocytogenes (11). In Sm mice infected with the Δ*hly* strain, the tag abundances in fecal samples remained fairly stable and closely resembled the input population over the course of 5 days (Fig. 4C and S3B). Animals infected with the Δ*hly* strain had no or few L. monocytogenes CFU recovered from their gallbladders, making assessment of pathogen population structures at this site infeasible.

10.1128/mBio.02948-19.3 FIG S3 STAMP analysis. C57BL/6 mice (*n* = 5) received Sm pretreatment and were infected with 10^8^ CFU of L. monocytogenes library containing 200 genetically tagged strains. (A) Relative tag abundance recovered from input and fecal pellets of mice infected with a wild-type strain 5 days postinfection. Tags are indicated by color. Panels represent individual mice. (B) Relative tag abundances recovered from input and fecal pellets of mice infected with a Δ*hly* strain 5 days postinfection. Tags are indicated by color. Panels represent individual mice. (C) Genetic relatedness relative to input over time. Details on genetic relatedness calculation are described in Materials and Methods. (D) Genetic relatedness of fecal pellets 5 days postinfection relative to input and gallbladder. A statistically significant difference is not indicated (*P* = 0.0746 by unpaired two-tailed test *t* test). Download FIG S3, PDF file, 1.0 MB. Copyright © 2019 Louie et al. 2019 Louie et al. This content is distributed under the terms of the Creative Commons Attribution 4.0 International license.

To quantify changes in population structures, we calculated a relatedness score based on changes in tag abundances found in feces 5 days postinfection compared to abundances found in the inoculum (Fig. 4D and S4C and D). The relatedness score of the Δ*hly* populations recovered from mice 5 days postinfection was 0.85, indicating that the population remained highly similar to the inoculum. In contrast, the relatedness score was 0.44 for the wild-type populations recovered from mice 5 days postinfection (Fig. 4D and S4C and D). Together, the absence of clonal enrichment in fecal samples recovered from infections with the Δ*hly* strain along with the enrichment of gallbladder-derived bacteria in infections with the wild-type strain support a model where LLO-dependent systemic spread leads to the establishment of a bacterial reservoir in the gallbladder which becomes the dominant bacteria population shed in feces.

## DISCUSSION

L. monocytogenes infection begins through the consumption of contaminated food, yet the i.v. infection of mice remains the most widely used *in vivo* model of L. monocytogenes pathogenesis. Here, we describe a noninvasive and genetically tractable mouse model of foodborne infection that leads to the development of intestinal pathology as well as systemic dissemination. During the course of infection, bacteria disseminated from the intestinal tract to systemic sites, including the spleen, liver, and gallbladder. Following the proliferation of a very small number of founding bacterial cells (often one) to high densities in the gallbladder (11), L. monocytogenes reentered the intestinal tract through the bile duct, and these bacteria, which had undergone replication at systemic sites, outcompeted their intestine-resident counterparts. L. monocytogenes lacking LLO or ActA proliferated in the intestines but was highly impaired in its capacity to complete this multiorgan trafficking circuit. Thus, the ability to complete its intracellular life cycle may confer an advantage in environmental dispersion for L. monocytogenes.

Compared to other small animal models of L. monocytogenes infection, the foodborne enterocolitis model presented here has several advantages. This model offers a system that utilizes the natural route of infection, the genetically tractable C57BL/6 mouse genetic background, and infectious doses as low as 100 CFU. These attributes facilitate experiments aimed at understanding L. monocytogenes biology in the intestinal environment, host factors involved in the initial phase of infection, and bacterial determinants of intestinal pathogenesis. In comparison to a previously described foodborne model (14), the enterocolitis model leads to more severe disease marked by weight loss, acute diarrhea, influx of immune cells into intestinal tissues, and fecal shedding of up to 10^9^ CFU/g. Notably, monitoring weight loss during infection is a fairly simple approach for quantifying disease severity. The high number of L. monocytogenes cells recovered from feces provides a wide dynamic range for characterizing factors involved in intestinal colonization. Given the very large number of C57BL/6 mutant mice and immunological tools available for L. monocytogenes infection in mice, the enterocolitis model is an attractive system for the study of mucosal immunology.

An important confounding feature of the enterocolitis model is the streptomycin pretreatment. Because streptomycin treatment alters the composition of the microbiota, this model will have limited use for probing interactions between L. monocytogenes and an intact microbiota. Furthermore, the downstream consequences of dysbiosis induced by streptomycin treatment, such as disruption of the hypoxic state within intestinal tissues (19), can complicate interpretations of findings from this model. Another potential limitation of the enterocolitis model is that internalin A (InlA)-dependent mechanisms of pathogenesis may not apply in this setting, because mouse E-cadherin and InlA have weak interactions (20, 21); however, dissemination in the enterocolitis model appears to follow the route described in the guinea pig model, where interactions between InlA and E-cadherin play a role (9). Despite these limitations, this model will aid our efforts to understand the molecular mechanisms of L. monocytogenes pathogenesis.

The roles of LLO and ActA during the systemic phase of L. monocytogenes infection have been extensively characterized, and absence of either virulence factor leads to severe attenuation in the i.v. mouse model (4, 5). The characterization of LLO and ActA in the development of enterocolitis presented here complements the existing literature on LLO and ActA in the intestinal setting. We report that infection with wild-type but neither Δ*hly* nor Δ*actA* L. monocytogenes resulted in intestinal inflammation, diarrhea, and weight loss. These observations are consistent with studies in germfree mice, where administration of a Δ*hly* strain did not trigger immune cell infiltration of intestinal tissues (22). Similarly, formerly germfree mice colonized with a Δ*actA* strain remained disease free for up to 90 days postinfection (23). Together, these findings suggest that the recruitment of immune cells to intestinal tissues and the subsequent development of pathology require the intact L. monocytogenes intracellular life cycle. In the enterocolitis model described in this study, both LLO and ActA were required for robust systemic dissemination. Similar findings have been reported in studies of germfree mice and in mice with intact microbiota (22, 24). This defect in systemic spread could be explained by a reduced capacity of the mutants to cross the intestinal barrier and/or poor survival at systemic sites, as previously observed in studies using the i.v. infection model (6–8).

Importantly, the differences in pathology and systemic dissemination between mice infected with wild-type, Δ*hly*, and Δ*actA* strains could not be explained by differences in the intestinal abundance of these strains. In the enterocolitis model, mice infected with wild-type and Δ*hly* strains shed comparable levels of L. monocytogenes in feces over the course of the study. Studies in a germfree setting and in the presence of an intact microbiota yielded similar findings for a Δ*hly* strain, suggesting that intestinal colonization does not require LLO and that luminal L. monocytogenes was not sufficient to cause disease (22, 24). Mice infected with the Δ*actA* strain shed almost 50-fold less L. monocytogenes than mice infected with the wild type at 5 days postinfection, which may be due to the role of ActA in bacterial aggregate formation during intestinal carriage (25). Alternatively, given the reported vaccination efficacy of the Δ*actA* strain (6, 26) and the late onset of the difference in fecal shedding, the potential contribution of the host adaptive immune response in controlling bacterial growth in the intestinal tract under these conditions warrants further investigation.

Successful enteric pathogens utilize strategies to subvert colonization resistance mechanisms imposed by the intestinal microbiota (27). Colonization resistance mechanisms are successful against incoming L. monocytogenes, as demonstrated by the effectiveness of streptomycin pretreatment in enabling the growth of L. monocytogenes in the intestine (18). However, it has been proposed that intestinal microbiota composition could represent an important risk factor for listeriosis, given that physiological conditions often associated with increased risk of severe listeriosis such as advanced age, pregnancy, and compromised immunity are also associated with changes to microbiota composition (28). Although the mechanisms by which the intestinal microbiota inhibits L. monocytogenes colonization remain unknown, members of the *Clostridiales* are sufficient for protection (18). Despite these defenses, L. monocytogenes overcomes colonization resistance and causes disease, yet the mechanisms by which this occurs remain incompletely defined. One strategy is seen in a subset of outbreak-associated L. monocytogenes strains that use a bacteriocin called listeriolysin S to directly limit competing bacteria in the intestine (29). As the infection progresses, gallbladder colonization could provide an alternative strategy that minimizes the need to directly compete with the intestinal microbiota. By replicating to densities as high as 10^7^ per gallbladder, the intestinal tract acts as a conduit for release into the environment, where L. monocytogenes can grow as a free-living saprophyte until encountering a suitable host. The experiments presented here do not directly address the mechanisms by which the gallbladder population gains an advantage over intestine-resident counterparts. Perhaps diarrhea during infection creates an environment in which incoming L. monocytogenes from the gallbladder displaces intestine-resident bacteria. Another possibility is that exposure to the gallbladder environment induces adaptive responses in L. monocytogenes that promote survival and/or growth in the intestines. Notably, another enteric facultative intracellular pathogen *Salmonella* Typhi has independently evolved a similar strategy. Presence of *S.* Typhi in the gallbladder has been linked to the development of asymptomatic carriers such as Mary Mallon, or “Typhoid Mary,” which play critical roles in transmission (30, 31).

The biphasic lifestyle of L. monocytogenes necessitates the maintenance of genes required for growth as a free-living saprophyte and those required for growth as an intracellular pathogen. Gaps remain in our understanding of how these two programs are maintained. Activation of virulence genes under soil-mimicking conditions leads to a loss in competitiveness (32). However, the *hly* gene which encodes LLO appears to be under purifying selection, because the ratio of nonsynonymous to synonymous substitutions (dN/dS) estimated for *hly* (dN/dS = 0.03674) is similar to that of core genes (dN/dS = 0.05353) (33). Additionally, of 57,820 isolates of L. monocytogenes from the environment, only 5 contained mutations in *hly* (33). In this study, an intraspecies competition developed between intestine-resident and gallbladder-derived bacteria, with the gallbladder-derived bacteria eventually becoming dominant in feces. This also occurs in animals with intact microbiota, suggesting that the intraspecies competition did not arise due to alterations in intestinal niches resulting from streptomycin treatment (11). Since very few L. monocytogenes cells ultimately seed the gallbladder (8, 10)—representing a severe population bottleneck—and gallbladder colonization following foodborne infection requires LLO and ActA, these within-host bottlenecks provide purifying selection to maintain the integrity of the virulence program and suggest that host association plays a key role in the life history of L. monocytogenes. Thus, our work offers insight into how evolutionary forces that promote the virulence of this facultative intracellular pathogen can promote its presence in the environment.

## MATERIALS AND METHODS

### Ethics statement.

This study was carried out in strict accordance with the recommendations in the Guide for the Care and Use of Laboratory Animals of the National Research Council of the National Academy of Sciences (34). All protocols were reviewed and approved by the Animal Care and Use Committee at the University of California, Berkeley (AUP-2016-05-8811).

### Bacterial strains and growth conditions.

All L. monocytogenes strains used in this study were derived from wild-type 10403S (Table 1) and propagated in filter-sterilized brain heart infusion (BHI) medium (BD) at 37°C with shaking and without antibiotics unless otherwise stated in Materials and Methods. Cell density was spectrophotometrically measured by optical density at a wavelength of 600 nm (OD_600_). Frozen bacterial stocks were stored at −80°C in BHI medium plus 40% glycerol. Culture medium supplements were used at the following concentrations: streptomycin at 200 μg/ml, chloramphenicol at 7.5 μg/ml, nalidixic acid at 15 μg/ml, LiCl at 6 mg/ml, and glycine at 6 mg/ml.

**TABLE 1:** Strains

Strain	Description	Reference or source
10403S	Wild type	40
DP-L4027	Δ*hly* (*DPL-3079*)-phage cured	35, 41
DP-L4029	Δ*actA* (*DPL-2161*)-phage cured	35, 42
DP-L6768	Barcoded wild-type library	This study
DP-L6769	Barcoded Δ*hly* library	This study
XL1	For vector construction	Stratagene
SM10	Conjugation strain	43

### Plasmid and strain construction.

Escherichia coli strains used in this study are listed in Table 1. For vector construction, plasmids were maintained in E. coli DH5α. Plasmids were introduced into L. monocytogenes by conjugation as previously described, using a donor E. coli SM10 and a compatible L. monocytogenes strain (35). Barcoded L. monocytogenes libraries were constructed as previously described using pTZ200.mix (11), a pooled plasmid library containing 200 unique barcodes assembled onto a pPL2 backbone which stably integrates into the L. monocytogenes chromosome (35).

### Mice.

C57BL/6 mice were purchased from the Jackson Laboratory and maintained under specific-pathogen-free conditions at the University of California, Berkeley animal facility. Sex- and age-matched controls were used in all experiments according to institutional guidelines for animal care. Unless otherwise specified, 8- to 12-week-old female mice were used for all experiments.

### L. monocytogenes*-*induced colitis.

Prior to infection, 5 mg/ml of streptomycin sulfate was added to the drinking water. After 32 h, mice were transferred to fresh cages, and chow was removed to initiate an overnight fast. Forty-eight hours after streptomycin was added to the water, mice were isolated, fed a 3-mm piece of bread with 3 μl of butter and an inoculum of L. monocytogenes in PBS, and returned to cages containing standard drinking water and chow. Following infection, stools were collected and homogenized in PBS by vortexing for 5 min at 4°C, and dilutions were plated. In instances where streptomycin was not sufficient to restrict growth of intestinal bacteria, plates were supplemented with nalidixic acid, LiCl, and glycine. To confirm the identity of colonies recovered from feces, PCR for *actA* was performed. To determine bacterial burden in organs, mice were euthanized and tissues were collected. Livers were homogenized in 10 ml, while ileum (distal third of small intestines), cecum, colon, mLN, and spleen were homogenized in 2 ml 0.1% IGEPAL CA-630 (Sigma). Gallbladders were homogenized in 2 ml PBS. Dilutions of homogenates were plated to enumerate CFU.

### STAMP.

Sequence tag-based analysis of microbial populations (STAMP) analysis was performed as previously described (11, 36). L. monocytogenes colonies were washed off plates, genomic DNA was extracted, and the region harboring the 30-bp barcodes was amplified using primer PLM30 and primer PLM6-P29 (see Table S1 in the supplemental material). The purified PCR products were combined in equimolar concentrations and sequenced on an Illumina MiSeq machine using primer PLM49. Reaper-12-340 was used to discard sequence reads with low quality (≤Q30) and trim the sequence following the barcode. The trimmed sequences were clustered with QIIME (version 1.6.0) using pick_otus.py with a sequence similarity threshold of 0.9. Genetic distance was estimated using the Cavalli-Sforza chord distance method (37) as described by Abel et al. (36). Genetic relatedness is 1 − genetic distance.

10.1128/mBio.02948-19.4 TABLE S1 Primers. Download Table S1, PDF file, 0.1 MB. Copyright © 2019 Louie et al. 2019 Louie et al. This content is distributed under the terms of the Creative Commons Attribution 4.0 International license.

### Gentamicin treatment.

Cecal tissues were cut longitudinally, washed with cold PBS, and incubated in RPMI (Gibco) containing 5% fetal calf serum, HEPES, l-glutamine, and 100 μg/ml gentamicin for 45 min at 37°C. Tissues were washed 6 times by placing the tissue into 10 ml PBS on a rotator at 4°C for 20 min. Tissues were homogenized in 2 ml 0.01% 0.1% IGEPAL CA-630, and dilutions were plated.

### Histology.

For hematoxylin and eosin staining, tissues were fixed in buffered 4% paraformaldehyde (PFA). Histology was performed by HistoWiz Inc. (Brooklyn, NY) using a standard operating procedure and fully automated workflow. Samples were processed, embedded in paraffin, and sectioned at 4 μm. After staining, sections were dehydrated and film coverslipped using a TissueTek-Prisma and Coverslipper (Sakura). Whole-slide scanning (×40 magnification) was performed on an Aperio AT2 (Leica Biosystems). Samples were then scored by a trained pathologist blinded to the treatment group for inflammation, edema, goblet cell loss, and epithelial integrity, as previously described (38, 39) with the following modifications to the scoring system for goblet cell loss (see Table S2). The average number of goblet cells per high-power field (×400 magnification) was determined from 10 different regions of the colon epithelium. Since healthy wild-type animals housed in our facilities had 18 (standard deviation [SD], ±4) goblet cells per field, the scoring system for goblet cell loss was adjusted accordingly. In inflamed sections, scoring was taken in regions most affected. Counts were obtained only from fields where all 4 layers of the intestine were present.

10.1128/mBio.02948-19.5 TABLE S2 Histopathology score methodology. Download Table S2, PDF file, 0.1 MB. Copyright © 2019 Louie et al. 2019 Louie et al. This content is distributed under the terms of the Creative Commons Attribution 4.0 International license.

### Statistical analysis.

Statistical analyses were carried out with GraphPad Prism software (version 7.0a). See figure legends for details.
