# Supplementary figures and images for: A Multiorgan Trafficking Circuit Provides Purifying Selection of Listeria monocytogenes Virulence Genes
Source: mBio. 2019 Dec 17;10(6):e02948-19. doi: 10.1128/mBio.02948-19 (PMC6918090; doi:10.1128/mBio.02948-19)

**A**

Wild type

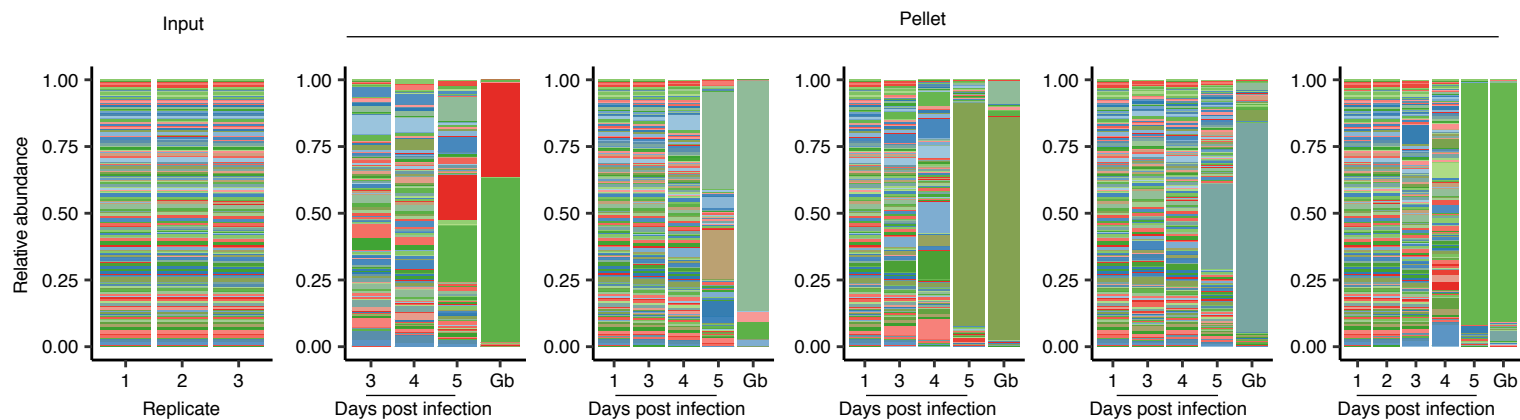**B** $\Delta hly$ 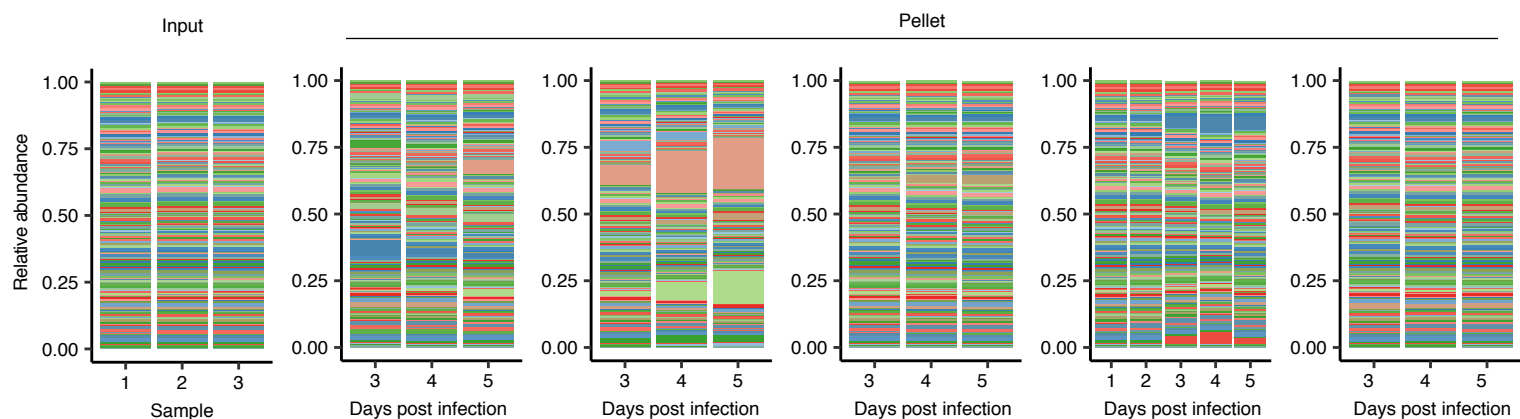**C**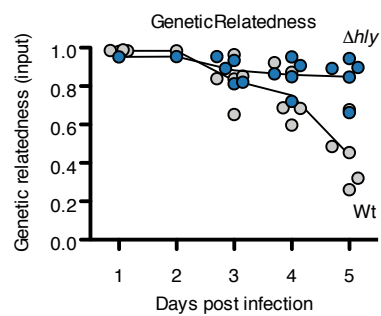**D**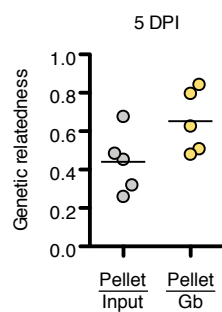

Supplement: FIG S3 [file mBio.02948-19-sf003.pdf]
