# Supplementary material for: A Multiorgan Trafficking Circuit Provides Purifying Selection of Listeria monocytogenes Virulence Genes
Source: mBio. 2019 Dec 17;10(6):e02948-19. doi: 10.1128/mBio.02948-19 (PMC6918090; doi:10.1128/mBio.02948-19)
Supplement: TABLE S1 [file mBio.02948-19-st001.pdf]

Table S1. Primers

Primer	Sequence (5'-3')	Description
PLM6	CAAGCAGAAGACGGCATACGAGATCGTGATGTGACTGGAGTTCAGACGTGTGCTCTTCCGATCTTGTCTCATGAGCGGATACA	Sequencing library construction
PLM7	CAAGCAGAAGACGGCATACGAGATACATCGGTGACTGGAGTTCAGACGTGTGCTCTTCCGATCTTGTCTCATGAGCGGATACA	Sequencing library construction
PLM8	CAAGCAGAAGACGGCATACGAGATGCCTAAGTGACTGGAGTTCAGACGTGTGCTCTTCCGATCTTGTCTCATGAGCGGATACA	Sequencing library construction
PLM9	CAAGCAGAAGACGGCATACGAGATTGGTCAGTGACTGGAGTTCAGACGTGTGCTCTTCCGATCTTGTCTCATGAGCGGATACA	Sequencing library construction
PLM10	CAAGCAGAAGACGGCATACGAGATCACTGTGTGACTGGAGTTCAGACGTGTGCTCTTCCGATCTTGTCTCATGAGCGGATACA	Sequencing library construction
PLM11	CAAGCAGAAGACGGCATACGAGATATTGGCGTGACTGGAGTTCAGACGTGTGCTCTTCCGATCTTGTCTCATGAGCGGATACA	Sequencing library construction
PLM12	CAAGCAGAAGACGGCATACGAGATGATCTGGTGACTGGAGTTCAGACGTGTGCTCTTCCGATCTTGTCTCATGAGCGGATACA	Sequencing library construction
PLM13	CAAGCAGAAGACGGCATACGAGATTCAAGTGTGACTGGAGTTCAGACGTGTGCTCTTCCGATCTTGTCTCATGAGCGGATACA	Sequencing library construction
PLM14	CAAGCAGAAGACGGCATACGAGATCTGATCGTGACTGGAGTTCAGACGTGTGCTCTTCCGATCTTGTCTCATGAGCGGATACA	Sequencing library construction
PLM15	CAAGCAGAAGACGGCATACGAGATAAGCTAGTGACTGGAGTTCAGACGTGTGCTCTTCCGATCTTGTCTCATGAGCGGATACA	Sequencing library construction
PLM16	CAAGCAGAAGACGGCATACGAGATGTAGCCGTGACTGGAGTTCAGACGTGTGCTCTTCCGATCTTGTCTCATGAGCGGATACA	Sequencing library construction
PLM17	CAAGCAGAAGACGGCATACGAGATTACAAGGTGACTGGAGTTCAGACGTGTGCTCTTCCGATCTTGTCTCATGAGCGGATACA	Sequencing library construction
PLM18	CAAGCAGAAGACGGCATACGAGATTGTTGACTGTGACTGGAGTTCAGACGTGTGCTCTTCCGATCTTGTCTCATGAGCGGATACA	Sequencing library construction
PLM19	CAAGCAGAAGACGGCATACGAGATACGGAACTGTGACTGGAGTTCAGACGTGTGCTCTTCCGATCTTGTCTCATGAGCGGATACA	Sequencing library construction
PLM20	CAAGCAGAAGACGGCATACGAGATTCTGACATGTGACTGGAGTTCAGACGTGTGCTCTTCCGATCTTGTCTCATGAGCGGATACA	Sequencing library construction
PLM21	CAAGCAGAAGACGGCATACGAGATCGGGACGGGTGACTGGAGTTCAGACGTGTGCTCTTCCGATCTTGTCTCATGAGCGGATACA	Sequencing library construction
PLM22	CAAGCAGAAGACGGCATACGAGATGTGCGGACGTGACTGGAGTTCAGACGTGTGCTCTTCCGATCTTGTCTCATGAGCGGATACA	Sequencing library construction
PLM23	CAAGCAGAAGACGGCATACGAGATCGTTTCACGTGACTGGAGTTCAGACGTGTGCTCTTCCGATCTTGTCTCATGAGCGGATACA	Sequencing library construction
PLM24	CAAGCAGAAGACGGCATACGAGATAAGGCCACGTGACTGGAGTTCAGACGTGTGCTCTTCCGATCTTGTCTCATGAGCGGATACA	Sequencing library construction
PLM25	CAAGCAGAAGACGGCATACGAGATTCCGAAACGTGACTGGAGTTCAGACGTGTGCTCTTCCGATCTTGTCTCATGAGCGGATACA	Sequencing library construction

Table S1. Primers

Primer	Sequence (5'-3')	Description
PLM26	CAAGCAGAAGACGGCATACGAGATTACGTACGGTGACTGGAGTT CAGACGTGTGCTCTTCCGATCTTGTCTCATGAGCGGATACA	Sequencing library construction
PLM27	CAAGCAGAAGACGGCATACGAGATATCCACTCGTGACTGGAGTT CAGACGTGTGCTCTTCCGATCTTGTCTCATGAGCGGATACA	Sequencing library construction
PLM28	CAAGCAGAAGACGGCATACGAGATATATCAGTGTGACTGGAGTT CAGACGTGTGCTCTTCCGATCTTGTCTCATGAGCGGATACA	Sequencing library construction
PLM29	CAAGCAGAAGACGGCATACGAGATAAAGGAATGTGACTGGAGTT CAGACGTGTGCTCTTCCGATCTTGTCTCATGAGCGGATACA	Sequencing library construction
PLM30	AATGATACGGCGACCACCGAGATCTACACTCTTTCCCTACACGAC GCTCTTCCGATCTTGTAAAACGACGGCCAG	Sequencing library construction
PLM49	ACGCTCTTCCGATCTTGTAAAACGACGGCCAGT	Sequencing primer
