# Supplementary material for: A Multiorgan Trafficking Circuit Provides Purifying Selection of Listeria monocytogenes Virulence Genes
Source: mBio. 2019 Dec 17;10(6):e02948-19. doi: 10.1128/mBio.02948-19 (PMC6918090; doi:10.1128/mBio.02948-19)
Supplement: TABLE S2 [file mBio.02948-19-st002.pdf]

Score	Inflammation (42)	Edema (42)	Goblet cells (43)	Epithelial integrity (43)
0	No pathological changes detectable in 10 high power fields at 400X (HPF)	No pathological changes detectable in 10 HPF	>18 / HPF	No pathological changes detectable in 10 HPF
1	Small multifocal mild lamina proprial and/or transepithelial leukocyte or submucosal accumulations	Mild segmental expansion of submucosa	10-17 / HPF	Epithelial desquamation and/or mild surface tattering
2	Moderate mucosal inflammation or submucosal extension	Moderate submucosal ± mucosal lamina propria expansion	1-9 / HPF	Erosion of the epithelial surface (gaps of 1 to 10 epithelial cells/lesion); and
3	Moderate to marked coalescing mucosal inflammation with prominent multifocal sub- mucosal extension +/– follicle formation (add 0.5 for moderate transmural inflammation)	Severe edema of mucosa and/or sub-mucosa	<1 /HPF	Epithelial ulceration (gaps of >10 epithelial cells/lesion; at this stage, there is generally granulation tissue below the epithelium).
4	Severe diffuse inflammation of mucosa, submucosa, deeper layers (transmural)	Transmural; very severe		
